# A PSO–ML-LSTM-based IMU state estimation approach for manipulator teleoperation

**DOI:** 10.3389/frobt.2025.1638853

**Published:** 2025-09-16

**Authors:** Renyi Zhou, Yuanchong Li, Aimin Zhang, Tie Zhang, Yisheng Guan, Zhijia Zhao, Shouyan Chen

**Affiliations:** 1 School of Electro-mechanical Engineering, Guangdong University of Technology, Guangzhou, China; 2 Advanced Technology Department, GAC R&D Center, Guangzhou Automobile Group Co. Ltd., Guangzhou, China; 3 School of Mechanical and Electrical Engineering, Guangzhou University, Guangzhou, China; 4 School of Mechanical and Automotive Engineering, South China University of Technology, Guangzhou, China

**Keywords:** state estimation, manipulator teleoperation, particle swarm optimization–modulated long short-term memory, master–slave mapping, cumulative errors

## Abstract

Manipulator teleoperation can liberate humans from hazardous tasks. Signal noise caused by environmental disturbances and the devices’ inherent characteristics may limit the teleoperation performance. This paper proposes an approach for inertial measurement unit (IMU) state estimation based on particle swarm optimization (PSO) and modulated long short-term memory (ML-LSTM) neural networks to mitigate the impact of IMU cumulative error on the robot teleoperation performance. A motion mapping model for the human arm and a seven-degree-of-freedom (7-DOF) robotic arm are first established based on global configuration parameters and a hybrid mapping method. This model is used to describe the impact of IMU cumulative error on the robot teleoperation performance. Subsequently, the IMU pose state estimation model is constructed using PSO and ML-LSTM neural networks. The initial data of multiple IMUs and handling handles are used for training the estimation model. Finally, comparative experiments are conducted to verify the performance of the proposed state estimation model. The results demonstrate that the PSO–ML-LSTM algorithm can effectively eliminate the impact of IMU cumulative errors on robot teleoperation.

## 1 Introduction

There are many environments and situations where robots are expected to replace or assist humans at the sites (Darvish et al., 2023; Liu et al., 2025). However, due to the limitations of AI techniques, fully autonomous solutions are still far from being able to generate natural and appropriate operational behaviors. Consequently, robot teleoperation is considered a reasonable solution for tasks in extreme environments, which can relieve human operators from potential hazards (Li S. et al., 2024). Various efforts have been made to deploy human senses, actions, and presence in remote locations. During the teleoperation, the robot is required to imitate human actions to perform highly dexterous tasks under limited information exchange. The priority of the robotic teleoperation system is to measure the kinematic and dynamic information of the human and transfer it to the robot’s movements for teleoperation. One of the key challenges lies in establishing precise motion mapping between the human operator and the robot.

To provide references for robotic motion, scholars have explored different technologies to measure human motion, including wearable inertial measurement unit (IMU)-based motion estimation (Penco et al., 2019; Škulj et al., 2021), vision-based motion capture (Tsitos and Dagioglou, 2022), exoskeleton-based motion measurement (Cheng et al., 2024), and EMG- and EEG-based motion intention estimation (Chen et al., 2023; Li H. et al., 2024). The performance of the vision-based capture approach is impacted by the occlusion and low portability of the setup, while the EMG- and EEG-based approaches have high requirements for the detection environment and equipment (Wang et al., 2023). In contrast, the IMU-based approach can estimate human motion without occlusion-related problems, which is more suitable for use in the field and other unstructured scenarios. However, gyroscopic drift of IMU tends to cause cumulative errors, which will gradually accumulate and amplify over time.

Scholars have attempted to improve IMU state estimation accuracy by deploying Kalman filtering and machine learning methods (Luo et al., 2025). Zhang et al. (2022) proposed a quadrotor state estimation method based on deep neural networks and a multi-sensor data fusion model. The IMU’s kinematic characteristics, the robot’s dynamic properties, and uncertainty representations are learned by training a cascaded network on real-world quadrotor flight data, the information of which is fused into a two-stage extended Kalman filter (EKF) framework for better estimation. Hosseinyalamdary (2018) proposed a combination of deep learning and Kalman filters for modeling to eliminate the system state estimation errors caused by IMU errors. Han et al. (2019) proposed a deep VIO algorithm that combines vision and IMU and uses a self-supervised end-to-end strategy to estimate system state. Kim et al. (2021) used one-dimensional convolutional neural networks to predict the desired velocity from the raw acceleration data to improve the accuracy of individual IMU state estimations. Luo et al. (2024) proposed a Kalman filtering and modulated long short-term memory (ML-LSTM)-based approach to estimate the state of the vehicle system. Xu et al. (2022) proposed a full-state estimation algorithm based on the error-state extended Kalman filter (ESEKF) framework, which can enable simultaneous state estimation and external calibration (POS–IMU and IMU–IMU), handheld platforms, quadrotor unmanned aerial vehicles (UAVs), and ground vehicles.

Furthermore, due to the structural differences between the human body and the robot, teleoperation control of robots requires consideration of the motion mapping between the human body and the robot. It can be categorized into motion mapping for the upper limbs, lower limbs, and the whole body. Upper-limb motion mapping typically involves mapping the Cartesian space movements of human limbs to the corresponding values of the robot’s limbs and then considering the robot’s constraints to solve the inverse kinematics problem by minimizing a cost function. A common approach is to establish motion mapping between the human wrist and the robot’s end-effector, which is known as configuration space retargeting (Wang et al., 2021). Zhao et al. (2023) proposed that the lower-priority elbow motion should be considered in the mapping process, which is important for delicate operations in constrained spaces. In reference to the above study, an IMU state estimation approach based on particle swarm optimization (PSO) and an ML-LSTM network is proposed to estimate the IMU error for robot teleoperation, while the human wrist and elbow motions are measured and imitated.

This study focuses on the human-robot posture mapping problem caused by multi-IMU drift errors in a remote sensing system. Unlike normal IMU drift issues, this problem requires establishing the spatial relationships among multiple IMUs on the human arm through kinematic modeling, leveraging their invariant spatial constraints to correct drift errors. The key contribution lies in proposing a lightweight, PSO-ML-LSTM-based online IMU calibration model that accounts for computational and temporal costs in data training. By fitting the model using early-stage IMU data (where drift is minimal), it enables real-time correction of subsequent drift-affected data. This method is used to establish the state and observation models for the teleoperation motion mapping, thereby enabling online calibration of the IMUs. The remainder of this study is structured as follows. Section II establishes the teleoperation mapping model and describes the background of the problem. Section III introduces the working principles of the algorithm. Section IV verifies the performance of the algorithm through comparative experiments. Section V summarizes the innovative methods proposed in this paper.

## 2 Robot teleoperation model and problem description

### 2.1 Robot teleoperation system

**FIGURE 1:**
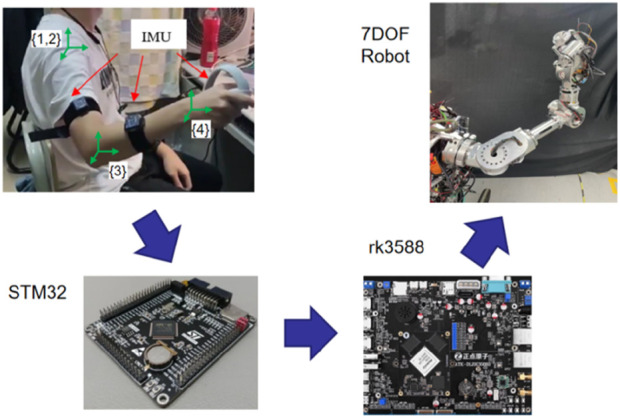
Robot teleoperation system.

The robot teleoperation system is based on two IMUs and an operating handle. The operating handle provides the position and orientation of the human hand, while the two IMUs, worn on the upper arm and forearm of the human body, are used to detect the motion state of the human arm. The data from the IMUs and operating handle are transmitted via Bluetooth to the STM32 for data acquisition and processing. Subsequently, the data are sent through a Wi-Fi module to the rk3588 processor for robot inverse kinematics and arm angle calculations, generating the trajectory of a seven-degree-of-freedom (7-DOF) robot. The system composition and data transmission process are illustrated in Figure 1.

### 2.2 Robot model

**FIGURE 2:**
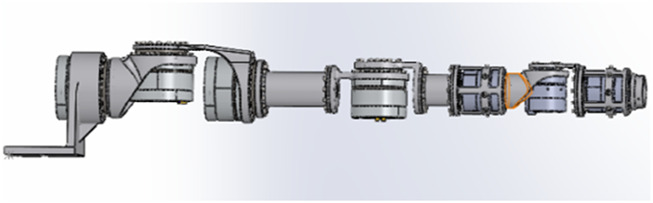
Robot configuration.

This paper adopts incremental position mapping and absolute attitude mapping. The kinematic model of the robot and the human body is established before determining the human–robot motion mapping. The 7-DOF robot structure is shown in Figure 2 and the robot DH parameters are provided in Table 1.

**TABLE 1:** Seven-DOF robot DH parameters.

i	$\alpha_{i-1}$	$a_{i-1}$	$d_{i}$	$\theta_{i}$
1	0°	0	0	$\theta_{r,1}$
2	−90°	0	0	$\theta_{r,2}$
3	90°	0	$d_{r,3}$	$\theta_{r,3}$
4	−90°	0	0	$\theta_{r,4}$
5	90°	0	$d_{r,5}$	$\theta_{r,5}$
6	−90°	0	0	$\theta_{r,6}$
7	90°	$a_{r,6}$	0	$\theta_{r,7}$

The robot kinematics model can be obtained by:

Trθ1,θ2,θ3,θ4,θ5,θ6,θ770=r11r12r13pxr21r22r23pyr310r320r33pz01=Rr70P7001. (1)

Here, P70=pr,x,pr,y,pr,z is the robot end-effector position.

To solve the problem of multiple solutions in the inverse solution of a redundant DOF manipulator, a unique solution can be obtained by introducing the global configuration parameter GCk. According to Faria et al. (2018), the spatial orientation of robotic arms is directly affected by GCk of the shoulder, elbow, and wrist.

**FIGURE 3:**
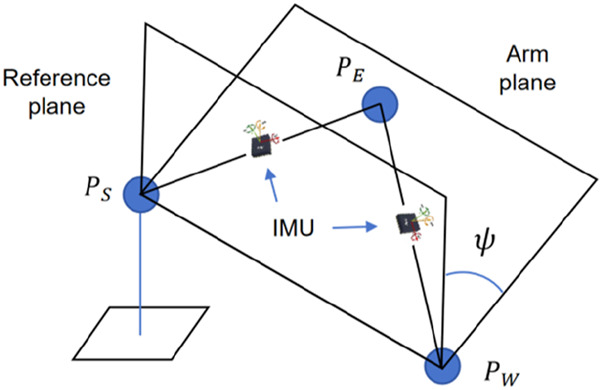
Representation of the arm angle ψ as the angle between the robot arm plane and the reference plane.

To address the global and local self-motion manifolds, two supplementary parameters are incorporated into the redundant robot inverse kinematics calculation, namely, global configuration (GC) and arm angle ψ . The global configuration is used to specify the branch of the inverse kinematics solutions for the global configuration manifold. The arm angle ψ indicates the elbow position in the redundancy circle, as shown in Figure 3. The global configuration parameter $GC_{k}$ is divided into three variables that represent the sign of the joint angle coordinates for the shoulder joint ( $GC_{2}$ ), the elbow joint ( $GC_{4}$ ), and the wrist joint ( $GC_{6}$ ). $GC_{k}$ is given as follows:

$$GC_{k}=\left\{\begin{array}{l} 1,\text{if}\theta_{k}\geq 0\\ -1,\text{if}\theta_{k}<0. \end{array}\right.,\forall k\in \left\{2,4,6\right\}.$$ (2)

In this paper, when the operator employs the right arm for teleoperation, the values are set as GC2 = 1, GC4 = 1, and GC6 = −1; conversely, when the left arm is utilized for teleoperation, the values are assigned as GC2 = −1, GC4 = −1, and GC6 = 1. Subsequently, a unique inverse solution can be derived based on the pose $T_{s,t_{1}}$ , the arm angle ψ , and the global configuration parameter GCk. Since θ_4 is not affected by the arm angle ψ , other angles can be obtained using the following formulas.

$$\begin{array}{ll} \theta_{1} & =\mathrm{atan}2\left(GC_{2}\left[a_{s22}\sin\psi +b_{s22}\cos\psi +c_{s22}\right],\right.\\ & \;GC_{2}\left.\left[a_{s12}\sin\psi +b_{s12}\cos\psi +c_{s12}\right]\right), \end{array}$$

$$\theta_{2}=GC_{2}\arccos\left(a_{s32}\sin\psi +b_{s32}\cos\psi +c_{s32}\right),$$

$$\begin{array}{ll} \theta_{3} & =\mathrm{atan}2\left(GC_{2}\left[-a_{s33}\sin\psi -b_{s33}\cos\psi -c_{s33}\right],\right.\\ & \;GC_{2}\left.\left[-a_{s31}\sin\psi -b_{s31}\cos\psi -c_{s31}\right]\right), \end{array}$$

$$\begin{array}{ll} \theta_{5} & =\mathrm{atan}2\left(GC_{6}\left[a_{w23}\sin\psi +b_{w23}\cos\psi +c_{w23}\right],\right.\\ & \;GC_{6}\left.\left[a_{w13}\sin\psi +b_{w13}\cos\psi +c_{w13}\right]\right), \end{array}$$

$$\theta_{6}=GC_{6}\arccos\left(a_{w33}\sin\psi +b_{w33}\cos\psi +c_{w33}\right),$$

$$\begin{array}{ll} \theta_{7} & =\mathrm{atan}2\left(GC_{6}\left[a_{w32}\sin\psi +b_{w32}\cos\psi +c_{w32}\right],\right.\\ & \;GC_{6}\left.\left[-a_{w31}\sin\psi -b_{w31}\cos\psi -c_{w31}\right]\right). \end{array}$$ (3)

### 2.3 Virtual human arm model

To construct a virtual human arm model, the coordinate system on the human arm is established as shown in Figure 1. Coordinate systems {1} and {2} are on the human shoulder joint, while coordinate system {3} is on the human elbow joint, and coordinate system {4} is established on the wrist. The DH parameterof virtual huma arm is shown in Table 2.

**TABLE 2:** Human arm DH parameters.

i	$\alpha_{i-1}$	$a_{i-1}$	$d_{i}$	$\theta_{i}$
1	0	0	0	$\theta_{h,1}$
2	90°	0	0	$\theta_{h,2}$
3	0	$a_{h,2}$	0	$\theta_{h,3}$
4	90°	$a_{h,3}$	0	$\theta_{h,4}$

Here, $a_{h,2}$ is the length of the upper arm and $a_{h,3}$ is the length of the lower arm. According to the homogeneous transformation formula, the elbow position can be derived as follows:

Ph30=ah,2ch,2ch,1ah,2ch,2sh,1ah,2sh,2. (4)

The wrist position is

Ph40=ch,1ah,3ch,23+ah,2ch,2sh,1ah,3ch,23+ah,2ch,2ah,3sh,23+ah,2sh,2. (5)

### 2.4 Master–slave motion relationship mapping

This paper employs a hybrid mapping approach, where position is mapped incrementally while orientation uses absolute mapping. The robot model and virtual human arm model are utilized to map the posture of both the human arm and wrist.

The positions of human wrist and robot can be obtained according to Equations 1, 5. The positions of the human wrist and robot end-effector are mapped incrementally. Assuming that the initial calibration position is $P_{m,t_{0}}=P_{h,t_{0}}$ and Ps,t0=Pr,t070 , the positions in relation to the master and slave ends can be expressed as follows:

$$P_{h,t_{1}}=P_{h,t_{0}}+∆P_{h,t_{1}},$$

$$P_{r,t_{1}}=P_{r,t_{0}}+∆P_{r,t_{1}},$$ (6)

$$∆P_{r,t_{1}}=K∆P_{h,t_{1}}.$$

Here, K is the mapping scale. The attitude of the human wrist and the robot end-effector are mapped absolutely:

$$∆R_{r,t_{1}}=∆R_{h,t_{1}}.$$ (7)

In order to ensure that the robot arm can adjust the attitude of the intermediate joint according to the requirements of the operator to achieve obstacle avoidance, the intermediate joint adopts absolute attitude mapping:

$$T_{h,t_{1}}=\left[P_{h,t_{1}},∆R_{h,t_{1}}\right],$$

$$T_{r,t_{1}}=\left[K∆P_{r,t_{1}},∆R_{r,t_{1}}\right].$$ (8)

According to Equations 6–8, the relationship mapping can be established. For ease of calculation, the arm angle ψ in this paper is defined as the angle between the arm and reference planes, where the arm plane is considered parallel to the axis $X_{1}$ and $X_{2}$ of the IMU on the upper and lower arms. To figure out the arm plane, the Euler angles of the two IMUs are converted into direction vectors. By defining the IMU Euler angles as $\left[\alpha_{1},\beta_{1},\gamma_{1}\right]$ and $\left[\alpha_{2},\beta_{2},\gamma_{2}\right]$ , the normal vector S of the arm plane can be obtained by the cross product of $X_{1}$ and $X_{2}$ .

$$S=X_{1}\times X_{2}=\left[\begin{array}{c} c\gamma_{1}*c\beta_{1}\\ s\gamma_{1}*c\beta_{1}\\ s\beta_{1} \end{array}\right]\times \left[\begin{array}{c} c\gamma_{2}*c\beta_{2}\\ s\gamma_{2}*c\beta_{2}\\ s\beta_{2} \end{array}\right].$$ (9)

Defining the reference plane as being perpendicular to the horizontal plane, the arm angle ψ can be obtained by dotting the normal vector S with the horizontal normal vector H .

$$\psi =arcos\left(S\cdot H\right).$$ (10)

The inverse kinematics can be obtained by substituting Equation 10 into Equation 2.

### 2.5 Problem description

When there is magnetic field interference in the operating environment, the magnetometer cannot be used to calibrate the IMU online, and the IMU cumulative error $\left[e_{1}\left(t\right),e_{2}\left(t\right)\right]$ will appear. In this case, the normal vector of the arm plane is $S^{\prime }\left(e_{1}\left(t\right),e_{2}\left(t\right)\right)$ , and the arm angle error is

$$\psi^{\prime }\left(t\right)=ar\cos\left(S^{\prime }\left(t\right)\cdot H\right).$$ (11)

**FIGURE 4:**
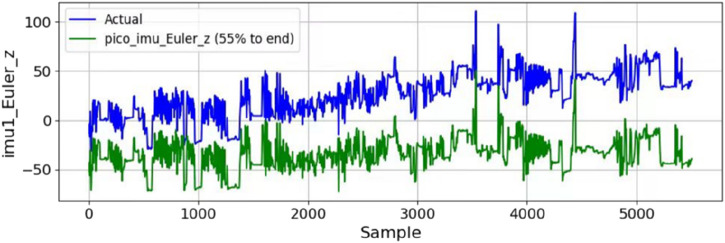
IMU cumulative error after 20 min.

To observe the drift problem, an IMU with magnetometer malfunction is bound with a high-precision IMU with magnetometer. A random arm motion is conducted to observe the drift of the low-cost IMU, while the high-precision IMU is regarded as a reference standard. The Figure 4 shows the IMU yaw angle without (blue line) and with the magnetometer after 20 min of various random motions. The final cumulative error is approximately 70°. It is worth noting that to perform online calibration of the IMU on the arm, accurate data are required as a reference point. Since the handle has its own vision-based calibration function, this paper assumes that the position data of the handle are accurate.

## 3 PSO–ML-LSTM-based IMU state estimation

**FIGURE 5:**
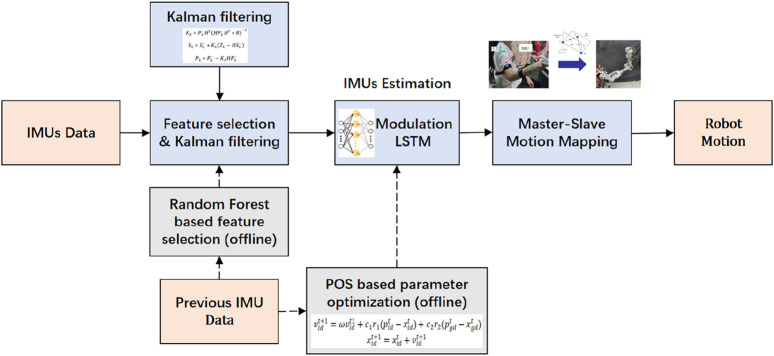
Process of PSO–ML-LSTM-based IMU state estimation.

In the case of magnetic field disturbance, the IMU cannot be self-calibrated by a magnetometer, and the cumulative error caused by drift will occur in the IMU. Therefore, it is necessary to estimate the IMU state online. This section introduces the PSO–ML-LSTM-based IMU state estimation approach. The whole process is shown in Figure 5.

### 3.1 Feature selection based on random forest

Considering the computation and time costs for online training, random forest-based feature selection is conducted to exclude unimportant feature data. Random forest-based feature selection combines the powerful representation capabilities of neural networks with the stability and nonlinear processing capabilities of random forest models. During the generation of each decision tree, the data subset and feature subset are randomly selected to enhance the robustness of the model and reduce overfitting. The input data for feature selection include the position and attitude of the operating handle, the attitude of IMUs, and the angular velocity and acceleration of IMUs. The feature selection results indicate that the position of the operating handle and the pitch and roll angle of IMUs have a significant impact on the IMUs’ yaw angle estimation.

### 3.2 Kalman filtering for data processing

In this paper, Kalman filtering is used to reduce IMU data bias and noise. To achieve this, the angular velocity measured by the gyroscope is used to predict the attitude and simultaneously model the slow change of zero offset:

$$x_{k}=Ax_{k-1}+Bu_{k}+w_{k}.$$ (12)

Here, A is the state transition matrix, $u_{k}$ is the angular velocity measured by the gyroscope, and $w_{k}$ is data noise. The process is started with the initiating states $x_{0}$ and $P_{0}$ . The prediction state ${\hat{x}}_{k}^{-}$ and error covariance $P_{k}^{-}$ are calculated as follows:

$${\hat{x}}_{k}^{-}=A{\hat{x}}_{k-1}+Bu_{k},$$

$$P_{k}^{-}=AP_{k-1}{\tilde{A}}^{T}+Q.$$ (13)

The Kalman gain $K_{k}$ and state are updated according to

$$K_{k}=P_{k}^{-}H^{T}{\left(HP_{k}^{-}H^{T}+R\right)}^{-1},$$

$${\hat{x}}_{k}={\hat{x}}_{k}^{-}+K_{k}\left(Z_{k}-H{\hat{x}}_{k}^{-}\right),$$

$$P_{k}=P_{k}^{-}-K_{k}HP_{k}^{-}.$$ (14)

Here, Q is the process noise covariance matrix, and R is the measurement noise covariance matrix.

### 3.3 PSO–ML-LSTM-based IMU state estimation

**FIGURE 6:**
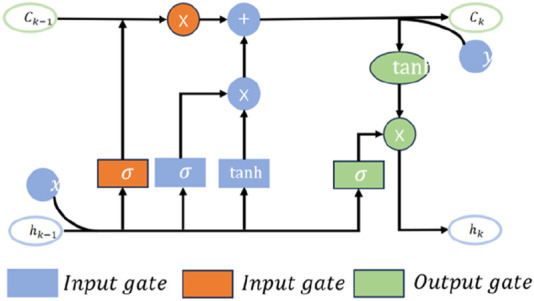
Structure of ML-LSTM.

The ML-LSTM neural network introduces the modulation gate into traditional LSTM to evaluate the importance of historical information at different times. It can improve the traditional memory mode and take the memory summation average value of each data segment as the standard memory, thus improving the model’s ability to recognize key information and the model’s ability to interpret. The structure of the ML-LSTM neural network is shown in Figure 6.

The forget gate of ML-LSTM is used to determine what information can pass through the memory unit and generate an $f_{k}$ according to the output value $h_{k-1}$ at the last moment and the current input value $x_{k}$ .

$$f_{k}=\sigma \left(W_{f}\cdot \left[h_{k-1},x_{k}\right]+b_{f}\right).$$ (15)

Here, $W_{f}$ is the weight, $b_{f}$ is the offset, and σ is the activation function sigmoid. To generate updated information, the values of $i_{k}$ and the new candidate ${\tilde{C}}_{k}$ will be calculated and added to the memory unit as a candidate value generated by the current layer.

$$i_{k}=\sigma \left(W_{i}\cdot \left[h_{k-1},x_{k}\right]+b_{i}\right),$$

$${\tilde{C}}_{k}=\tanh\left(W_{C}\cdot \left[h_{k-1},x_{k}\right]+b_{C}\right).$$ (16)

The memory cells are updated by

$${C_{k}}^{\prime }=f_{k}\cdot C_{k-1}+i_{k}\cdot {\tilde{C}}_{k}.$$ (17)

The modulation gate sums the memory information and calculates the mean *E*.

$$C_{k}=E=\sum_{1}^{m}\;\frac{{C_{k,m}}^{\prime }}{m}.$$ (18)

Here, m is the number of data segments. The model output $h_{k}$ can be obtained as follows:

$$h_{k}=\sigma \left(W_{o}\cdot \left[h_{k-1},x_{k}\right]+b_{o}\right)\cdot \tanh\left(C_{k}\right).$$ (19)

Then, the PSO algorithm is run offline using previous data to optimize the neuron number $n_{nn}$ and learning rate $l_{r}$ of ML-LSTM. The initial position and speed of the particles are first randomly generated. The fitness evaluation of each particle solution is conducted. The historical best position ( $p_{id}^{t}$ ) and the global best position ( $p_{gd}^{t}$ ) for each particle are then recorded. The speed and position of each particle according to $p_{id}^{t}$ and $p_{gd}^{t}$ are updated to calculate the neuron number and learning rate according to the following equation.

$$v_{id}^{t+1}=\omega v_{id}^{t1}+c_{1}r_{1}\left(p_{id}^{t}-x_{id}^{t}\right)+c_{2}r_{2}\left(p_{gd}^{t}-x_{gd}^{t}\right),$$

$$x_{id}^{t+1}=x_{id}^{t}+v_{id}^{t+1}.$$ (20)

**FIGURE 7:**
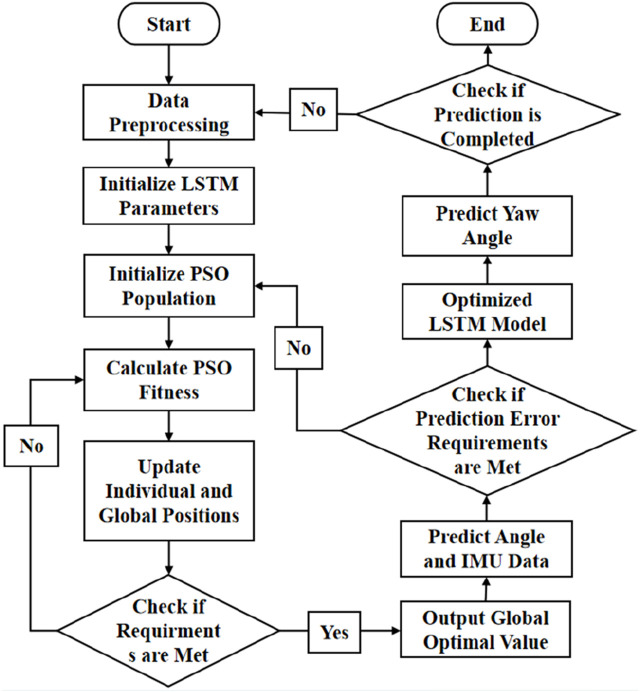
Process of PSO.

Here, $v_{id}^{t+1}$ and $x_{id}^{t+1}$ can be represented by $v_{lr}$ and $v_{nn}$ and $l_{r}$ and $n_{nn}$ . The above process will be repeated until the number of iterations is reached or an optimal solution is found. The PSO flow chart is shown in Figure 7.

### 3.4 Evaluation index

To evaluate the performance of the IMU state estimation model, three evaluation indexes are used, including the root mean square error (RMSE), mean absolute error (MAE), mean bias error (MBE), and R-squared $R^{2}$ .

$$MAE\left(X,h\right)=\frac{1}{m}\sum_{i=1}^{m}\;\left|h\left(x_{i}\right)-y_{i}\right|,$$ (21)

$$RMSE\left(X,h\right)=\sqrt{\frac{1}{m}\sum_{i=1}^{m}\;{\left(h\left(x_{i}\right)-y_{i}\right)}^{2}},$$ (22)

$$\text{MBE}=\frac{1}{n}\sum_{i=1}^{n}\;\left(y_{i}-{\hat{y}}_{i}\right)$$ (23)

$$R^{2}=1-\frac{\sum_{i=1}^{n}\;{\left(h\left(x_{i}\right)-y_{i}\right)}^{2}}{\sum_{i=1}^{n}\;{\left(h\left({\overset{¯}{x}}_{i}\right)-y_{i}\right)}^{2}}$$ (24)

## 4 Experiment and discussion

In this paper, the IMU estimation model is implemented online using LibTorch, which is integrated into the rk3588 processor. The dataset is split into training and validation sets with a ratio of 2:8. The random forest is first used for feature selection. and the particle swarm optimization is used to optimize the number of neurons and the learning rate of the ML-LSTM model before online estimation to reduce data requirement for model training and prediction. The search bounds for the number of neurons are set to [50, 200], while the learning rate is bounded within [1e-5, 1e-1]. The PSO hyperparameters are configured as follows: population size = 10, maximum iterations = 20, decay factor = 0.5, contraction–expansion coefficient = 1.0, and random seed = 42. The Adam optimizer is used for network training, where the training batch size is 64 and the number of iterations is 1,000. To confirm the validity of the proposed method, we compared it with other models, including Gaussian process regression (GPR) based on the radial basis kernel function (Ouyang et al., 2023), BP neural network based on the Levenberg–Marquardt algorithm (Hua et al., 2023), and neural network based on PSO–LSTM (Xiao et al., 2024). In order to observe the performance of the estimation models, the IMU data of 30 min without and with magnetometer calibration are compared with the IMU data estimated by the model mentioned above.

### 4.1 Levenberg–Marquardt-BP-based IMU estimation

**FIGURE 8:**
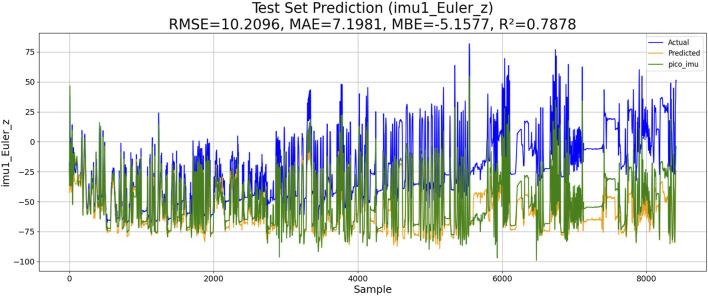
Performance of LM-BP-based IMU estimation.

The Levenberg–Marquardt-BP neural network can accelerate the training process and is suitable for complex nonlinear regression problems. The goal of this algorithm is to adjust the network weight by minimizing the error function. The algorithm implementation process is as follows: 1) the gradient of the loss function is calculated. 2) The weights are updated according to the Levenberg–Marquardt algorithm. 3) Iterations are repeated until convergence or the maximum number of iterations is reached. The model calculation results are given below, where the forecast results are colored yellow, the drift data are colored blue, and the correct data are colored green. The performance of LM-BP-based IMU estimation is shown in Figure 8.

### 4.2 K-means-RBF-GPR-based IMU estimation

**FIGURE 9:**
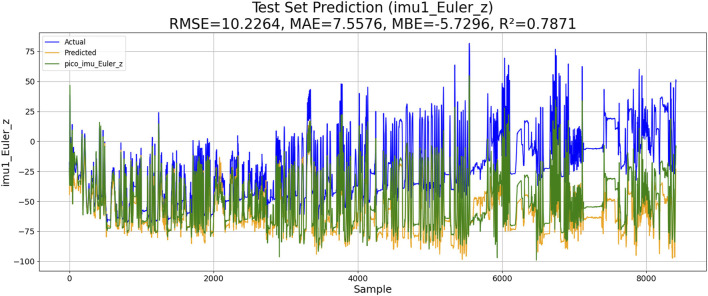
Performance of K-means-RBF-GPR-based IMU estimation.

Gaussian process regression is a powerful non-parametric Bayesian regression method. It uses kernel functions to capture complex relationships of data by mapping the input space to a high-dimensional feature space. The cluster center of K-means (KM) is selected as the input feature point. The similarity between these feature points is calculated using the RBF kernel function. The model parameters are optimized by maximizing the posterior probability or minimizing the negative log-likelihood. The performance of KM-RBF-GPR-based IMU estimation is shown in Figure 9.

### 4.3 PSO–ML-LSTM-based IMU estimation

**FIGURE 10:**
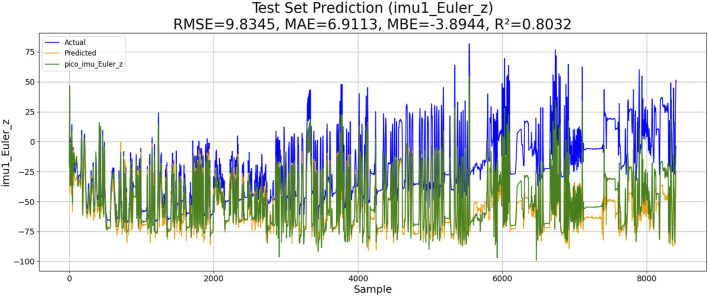
Performance of PSO–LSTM-based IMU estimation.

**FIGURE 11:**
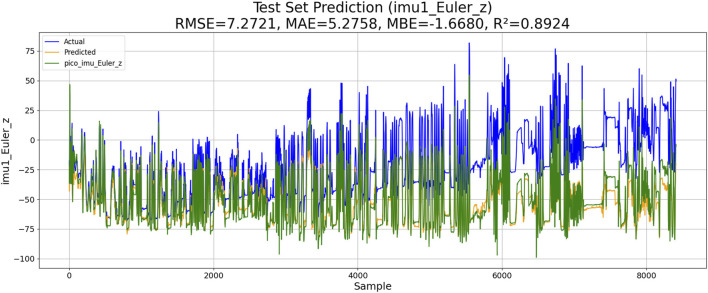
Performance of PSO–ML-LSTM-based IMU estimation.

Two experiments are conducted, including the PSO–LSTM- and the PSO–ML-LSTM-based IMU estimations. The performances of both models are shown in Figures 10, 11.

The results of the experiments are shown in Table 3. Although KM-RBF-GPR slightly outperforms LM-BP in terms of RMSE and MAE, it exhibits a larger bias (MBE) and relatively lower goodness of fit. The performance of PSO–LSTM is superior to that of KM-RBF-GPR, particularly showing an improvement in the goodness of fit (*R* ^2^), but it still cannot surpass that of PSO–ML-LSTM. The RMSE of PSO–ML-LSTM is 8.3116, which is 1.9 units lower than that of LM-BP and 1.5 units lower than the RMSE of PSO–LSTM. The MAE and *R* ^2^ of PSO–ML-LSTM are 6.1617 and 0.8594, indicating that PSO–ML-LSTM has no significant systematic bias and fits the data well. The average cumulative error of PSO–ML-LSTM is approximately 2.8°, which is significantly better than that of the other three models.

**TABLE 3:** Performance comparison.

Method	RMSE	MAE	MBE	$R^{2}$	Average cumulative error
*LM-BP*	10.2096	7.1981	−5.157	0.7878	3.2°
*KM-RBF-GPR*	10.2264	10.5973	−5.7296	0.7871	3.2°
*PSO–LSTM*	9.8345	6.9113	−3.8944	0.8032	3.1°
*PSO–ML-LSTM*	7.2721	5.2758	−1.6680	0.8924	2.8°

### 4.4 Ablation study

**FIGURE 12:**
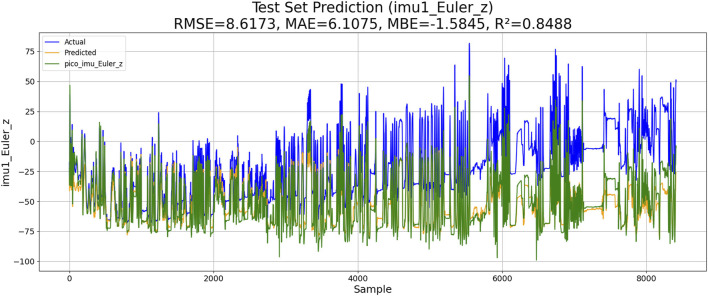
Performance of PSO–ML-LSTM without random forest feature selection.

**FIGURE 13:**
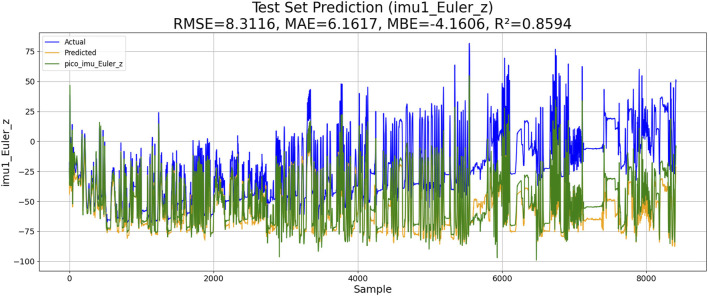
The performance of PSO-ML-LSTM without KF.

The ablation studies are conducted to evaluate the effects of the Kalman filter and random-forest feature selection. The results are shown in the Figures 12, 13. Compared with the result shown in figure, the results shown in Figure 8 indicate that the use of random-forest feature selection and Kalman filter can achieve a better performance of RMSE, MAE, MBE and *R* ^2^.

### 4.5 Robot remote control experiment

**FIGURE 14:**
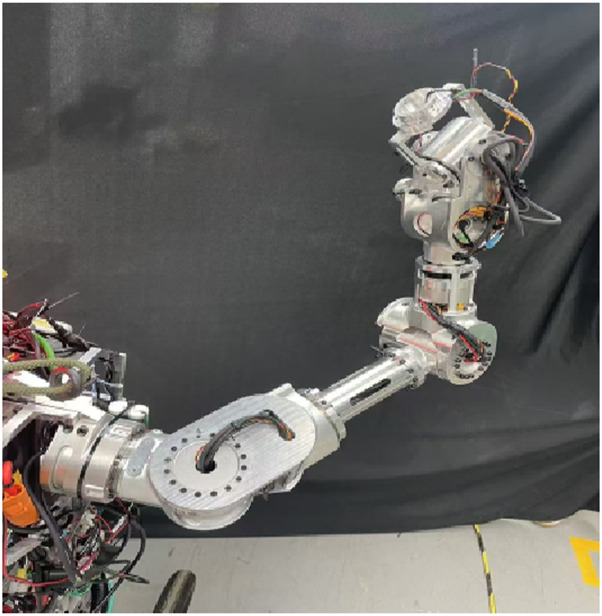
Seven-DOF manipulator.

**FIGURE 15:**
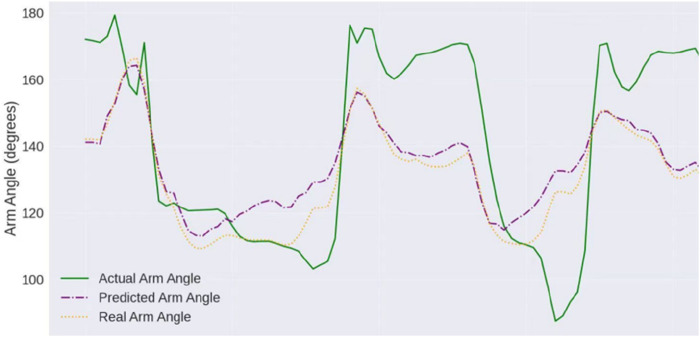
Robot arm angle under remote control.

A manipulator teleoperation experiment was conducted. By comparing the arm angles of the manipulator before and after IMU error compensation, the effectiveness of the proposed method was verified. The manipulator used was the 7-DOF manipulator described in the previous section, whose structure is shown in Figure 14. Figure 15 shows the arm angle conditions after 20 min, including 1) the manipulator’s arm angle without compensation (green curve), 2) the arm angle of the human operator (yellow curve), and 3) the manipulator’s arm angle using the proposed method (red curve). Without IMU error correction, the deviation between the manipulator’s arm angle and the operator’s arm angle is approximately 30°, and the deviation exhibits a nonlinear variation. After applying the proposed approach, the arm angle deviation is significantly reduced. The maximum deviation decreased from 30° to 10°, and the variation trends followed similar patterns.

**FIGURE 16:**
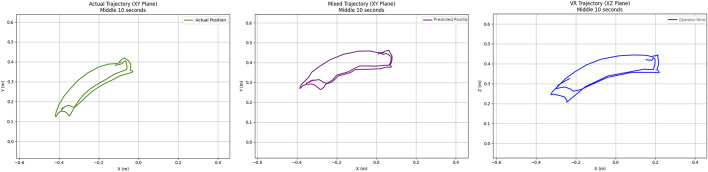
Robot end-effector position under remote control.

Figure 16 shows the robot end-effector position, including 1) the actual end-effector position without compensation (green curve), 2) the operator wrist position (blue curve), and 3) the predicted position of the end-effector using the proposed approach (purple curve). Without IMU error correction, the position deviation between the end-effector position and the operator’s wrist is approximately 0.25 m, and the angle of the end-effector differs significantly from that of the operator’s wrist. After applying the proposed approach, the position deviation is reduced to 0.13 m, and the end-effector’s angle closely matches the operator’s wrist.

## 5 Conclusion

This paper proposes a PSO-based modulated LSTM for online estimation of the IMU state in robot teleoperation. Experimental results show that the proposed method can effectively estimate the true attitude of the IMU, thus reducing the cumulative error of the IMU and the absolute error in teleoperation. However, the proposed method still has some limitations. For example, there is room for improvement in the RMSE and cumulative error.

## Data Availability

The raw data supporting the conclusions of this article will be made available by the authors, without undue reservation.
